# Supplementary material for: SREBP modulates the NADP+/NADPH cycle to control night sleep in Drosophila
Source: Nat Commun. 2023 Feb 20;14:763. doi: 10.1038/s41467-022-35577-8 (PMC9941135; doi:10.1038/s41467-022-35577-8)
Supplement: Supplementary file 1 — Supplementary Information [file 41467_2022_35577_MOESM1_ESM.pdf]

Supplementary Information

SREBP modulates the NADP$^+$/NADPH cycle to control night sleep in *Drosophila*

Vittoria Mariano, Alexandros K. Kanellopoulos, Giuseppe Aiello, Adrian C. Lo, Eric Legius, Tilmann Achsel, and Claudia Bagni

Supplementary Fig. 1

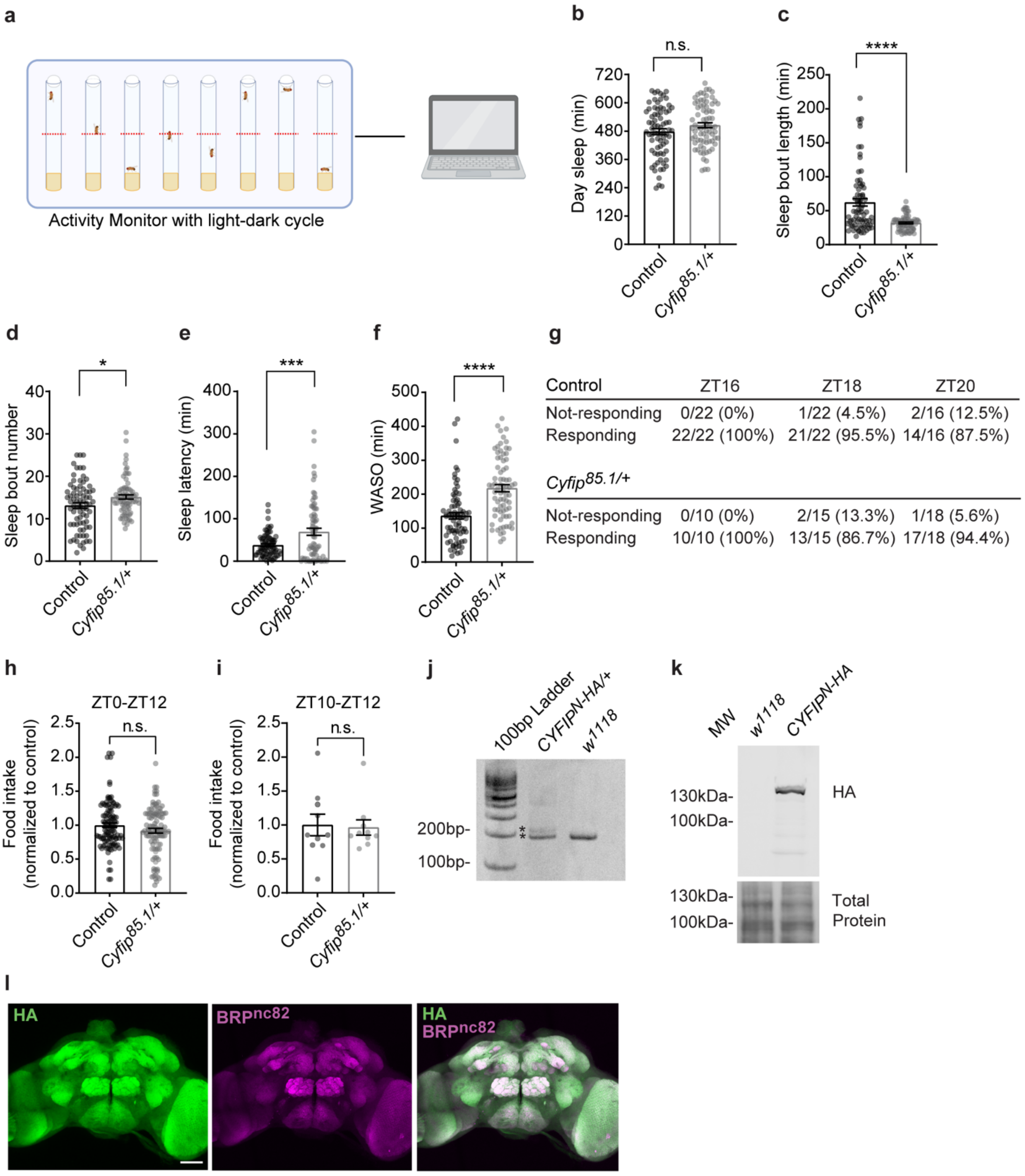

Supplementary Fig. 1 *Cyfip* regulates the quantity and quality of night-time sleep.

a Schematic of the *Drosophila* Activity Monitoring (DAM) System by Trikinetics. Created with BioRender.com. **b-f** Quantification of daytime sleep (**b**), night sleep bout length (**c**), night sleep bout number (**d**), night sleep latency (**e**), and wake after sleep onset (WASO) during night-time sleep (**f**) in control (n = 75) and $Cyfip^{85.1/+}$ (n = 75) flies. Two-tailed unpaired Student's t-test, (**b**) n.s. = not significant, $p = 0.0977$, (**c**) ****$p < 0.0001$, (**d**) *$p = 0.0168$, (**e**) ***$p = 0.0005$, (**f**) ****$p < 0.0001$. Mean ± S.E.M. **g** Number and percentage of flies that did not respond to light stimuli (Not-responding) and flies that woke up after light stimulus (Responding). Shown are the data for control and $Cyfip^{85.1/+}$ flies, and three time points when the stimulus was provided (ZT16, ZT18 and ZT20). Two-sided Fisher's exact test between Responding and Not-responding control and $Cyfip^{85.1/+}$, p = n.s. at ZT16, ZT18 and ZT20. **h-i** Quantification of food consumption during the light-on period (ZT0-ZT12) in $Cyfip^{85.1/+}$ (n = 108) and control (n = 108) flies (**h**) and just before light-off period (ZT10-ZT12) in $Cyfip^{85.1/+}$ (n = 10) and control (n = 10) flies (**i**). Two-tailed unpaired Student's t-test, n.s. = not significant. Mean ± S.E.M. **j** Representative image of PAGE in $CYFIP^{N\text{-}HA/+}$ flies after PCR genotyping of the region surrounding the HA tag insertion (see also Methods). Data are from one experiment with n = pool of 5 flies. Lower "*" indicates the wild-type and the upper "*" the HA-KI allele. **k** Representative Western blot from w^{1118} and $CYFIP^{N\text{-}HA}$ flies stained with an anti-HA antibody and for total protein. n = pool of 15 fly heads. Samples were analyzed in technical duplicates. **l** Representative images of immunohistochemistry in whole brain of $\text{CYFIP}^{\text{N-HA}}$ flies detecting anti-HA (green), anti-BRP^{nc82} (purple) and a merged image. Scale bar = 50 µm. A minimum of 5 fly brains were analyzed. Source data are provided as a Source Data file.

Supplementary Fig. 2

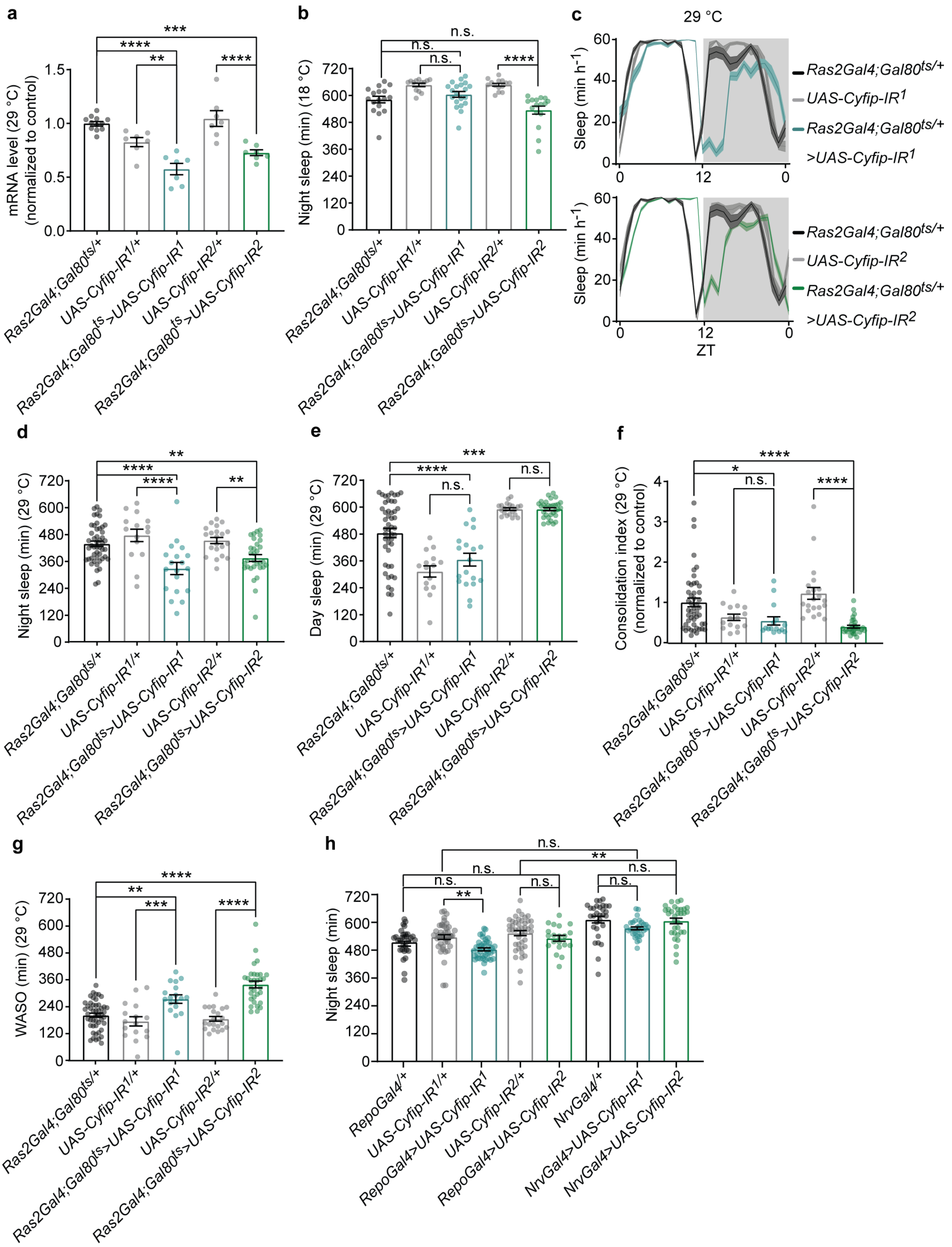

Supplementary Fig. 2 Neuronal expression of *Cyfip* is relevant for night-time sleep.

a RT-qPCR to assess the level of *Cyfip* mRNA normalized to *rpl32* mRNA. *Ras2Gal4;TubGal80*$^{ts/+}$ (n = 12), *UAS-Cyfip-IR*$^{1/+}$ (n = 7), *Ras2Gal4;TubGal80*^{ts}*>UAS-Cyfip-IR*^{1} (n = 7), *UAS-Cyfip-IR*$^{2/+}$ (n = 7) and *Ras2Gal4;TubGal80*^{ts}*>UAS-Cyfip-IR*^{2} (n = 7). n = independent samples, pool of 15 fly heads. One-way ANOVA and Sidak's multiple comparison test, **p = 0.0018, ***p = 0.001, ****p < 0.0001. Mean ± S.E.M. **b** Night-time sleep amount in pan-neuronal *Cyfip* knockdown at 18 °C. *Ras2Gal4;TubGal80*$^{ts/+}$ (n = 17), *UAS-Cyfip-IR*$^{1/+}$ (n = 13), *Ras2Gal4;TubGal80*^{ts}*>UAS-Cyfip-IR*^{1} (n = 20), *UAS-Cyfip-IR*$^{2/+}$ (n = 14) and *Ras2,Gal4;TubGal80*^{ts}*>UAS-Cyfip-IR*^{2} (n = 18) flies. Kruskal-Wallis test and Dunn's multiple comparisons test, ****p < 0.0001. Mean ± S.E.M. **c** Representative sleep profiles of *Ras2Gal4;TubGal80*$^{ts/+}$ (n = 20), *UAS-Cyfip-IR*$^{1/+}$ (n = 20), *Ras2Gal4;TubGal80*^{ts}*>UAS-Cyfip-IR*^{1} (n = 17) (upper panel) and *RasGal4;TubGal80*$^{ts/+}$ (n = 20), *UAS-Cyfip-IR*$^{2/+}$ (n = 20) and *Ras2Gal4;TubGal80*^{ts}*>UAS-Cyfip-IR*^{2} (n = 21) flies (lower panel). Mean ± S.E.M. **d** Night sleep amount in pan-neuronal *Cyfip* knockdown at 29 °C. *Ras2Gal4;TubGal80*$^{ts/+}$ (n = 48), *UAS-Cyfip-IR*$^{1/+}$ (n = 15), *Ras2Gal4;TubGal80*^{ts}*>UAS-Cyfip-IR*^{1} (n = 19), *UAS-Cyfip-IR*$^{2/+}$ (n = 21) and *Ras2Gal4;TubGal80*^{ts}*>UAS-Cyfip-IR*^{2} (n = 31) flies. One-way ANOVA and Sidak's multiple comparisons test. *Ras2Gal4;TubGal80*$^{ts/+}$ vs. *Ras2Gal4;TubGal80*^{ts}*>UAS-Cyfip-IR*^{2} **p = 0.009; *Ras2Gal4;TubGal80*^{ts}*>UAS-Cyfip-IR*^{2} vs. *UAS-Cyfip-IR*$^{2/+}$ **p = 0.0083; for the other comparisons ****p < 0.0001. Mean ± S.E.M. **e-g** Day sleep amount (**e**) Consolidation index (**f**) and WASO (**g**) in pan-neuronal *Cyfip* knockdown at 29 °C. In (e) and (g), *Ras2Gal4;TubGal80*$^{ts/+}$ (n = 48), *UAS-Cyfip-IR*$^{1/+}$ (n = 15), *Ras2Gal4;TubGal80*^{ts}*>UAS-Cyfip-IR*^{1} (n = 19), *UAS-Cyfip-IR*$^{2/+}$ (n = 21) and *Ras2Gal4;TubGal80*^{ts}*>UAS-Cyfip-IR*^{2} (n = 31) flies. In (f) *Ras2Gal4;TubGal80*$^{ts/+}$ (n = 48), *UAS-Cyfip-IR*$^{1/+}$ (n = 16), *Ras2Gal4;TubGal80*^{ts}*>UAS-Cyfip-IR*^{1} (n = 15), *UAS-Cyfip-IR*$^{2/+}$ (n = 21) and *Ras2Gal4;TubGal80*^{ts}*>UAS-Cyfip-IR*^{2} (n = 31) flies. One-way ANOVA and Sidak's multiple comparison test, (e) ***p = 0.0002, ****p < 0.0001; (f) *p = 0.0267, ****p < 0.0001; (g) **p = 0.0017, ***p = 0.0004, ****p < 0.0001. n.s. = not significant. Mean ± S.E.M. **h** Night sleep in *RepoGal4*$^{/+}$ (n = 30), *UAS-Cyfip-IR*$^{1/+}$ (n = 42), *RepoGal4>UAS-*

Cyfip-IR^{1} (n = 39), *UAS-Cyfip-IR*$^{2/+}$ (n = 43), *RepoGal4>UAS-Cyfip-IR*^{2} (n = 20), *NrvGal4*$^{/+}$ (n = 30), *NrvGal4>UAS-Cyfip-IR*^{1} (n = 32), *NrvGal4>UAS-Cyfip-IR*^{2} (n = 32) flies. One-way ANOVA and Sidak's multiple comparisons test, *UAS-Cyfip-IR*$^{1/+}$ vs. *RepoGal4>UAS-Cyfip-IR*^{1} $^{**}p = 0.001$; *UAS-Cyfip-IR*$^{2/+}$ vs. *NrvGal4>UAS-Cyfip-IR*^{2} $^{**}p = 0.0025$; for the other comparisons n.s. = not significant. Mean ± S.E.M. Source data are provided as a Source Data file.

Supplementary Fig. 3

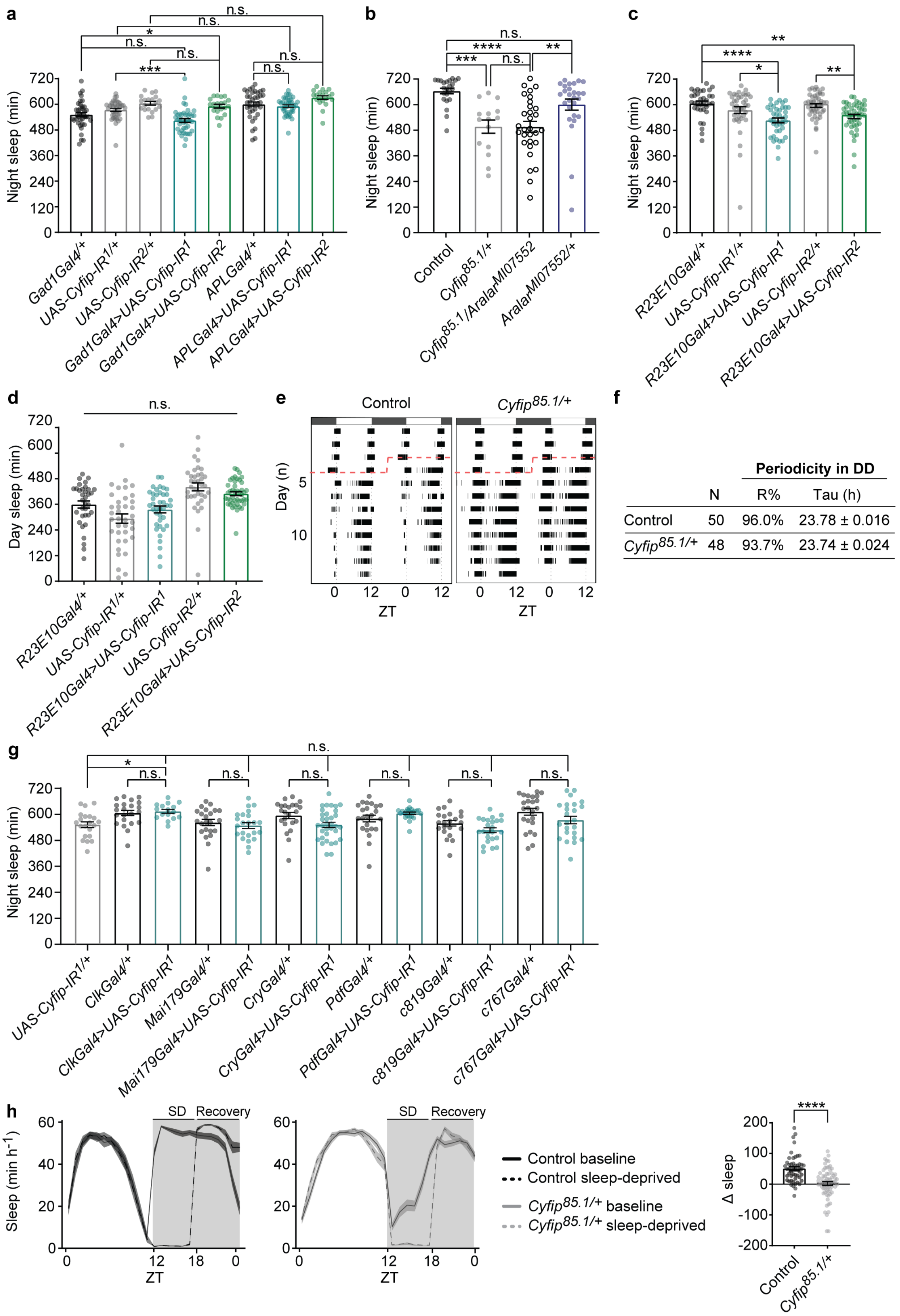

f

	Periodicity in DD		
	N	R%	Tau (h)
Control	50	96.0%	23.78 ± 0.016
Cyfip$^{85.1/+}$	48	93.7%	23.74 ± 0.024

Supplementary Fig. 3 *Cyfip*$^{85.1/+}$ night sleep is dependent on sleep homeostasis mechanisms.

a Night sleep in *APLGal4*$^{/+}$ (n = 36), *UAS-Cyfip-IR*$^{1/+}$ (n = 38), *UAS-Cyfip-IR*$^{2/+}$ (n = 20), *Gad1Gal4>UAS-Cyfip-IR*^{1} (n = 40), *Gad1Gal4>UAS-Cyfip-IR*^{2} (n = 20); *Gad1Gal4*$^{/+}$ (n = 40), *APLGal4>UAS-Cyfip-IR*^{1} (n = 40), *APLGal4>UAS-Cyfip-IR*^{2} (n = 20) flies. *p = 0.0403, ***p = 0.0003. Mean ± S.E.M. **b** Night sleep in control (n = 23), *Cyfip*$^{85.1/+}$ (n = 15), *Cyfip*$^{85.1}$*/Aralar*^{MI07552} (n = 30) and *Aralar*$^{MI0755/+}$ (n = 27) flies. **p = 0.0062, ***p = 0.0003, ****p < 0.0001. Mean ± S.E.M. **c** Night sleep in *R23E10Gal4*$^{/+}$ (n = 34), *UAS-Cyfip-IR*$^{1/+}$ (n = 39), *UAS-Cyfip-IR*$^{2/+}$ (n = 48), *R23E10Gal4>UAS-Cyfip-IR*^{1} (n = 39), *R23E10Gal4>UAS-Cyfip-IR*^{2} (n = 45) flies. *UAS-Cyfip-IR*$^{1/+}$ vs. *R23E10Gal4>UAS-Cyfip-IR*^{1} *p = 0.0159; *R23E10Gal4*$^{/+}$ vs. *R23E10Gal4>UAS-Cyfip-IR*^{2} **p = 0.0058; *R23E10Gal4>UAS-Cyfip-IR*^{2} vs. *UAS-Cyfip-IR*$^{2/+}$ **p = 0.0033; *R23E10Gal4*$^{/+}$ vs. *R23E10Gal4>UAS-Cyfip-IR*^{1} ****p < 0.0001. Mean ± S.E.M. **d** Day sleep in *R23E10Gal4*$^{/+}$ (n = 34), *UAS-Cyfip-IR*$^{1/+}$ (n = 37), *UAS-Cyfip-IR*$^{2/+}$ (n = 36), *R23E10Gal4>UAS-Cyfip-IR*^{1} (n = 39), *R23E10Gal4>UAS-Cyfip-IR*^{2} (n = 44) flies. One-way ANOVA. n.s. = not significant. Mean ± S.E.M. **e** Representative double-plotted actograms of control (n = 20) and *Cyfip*$^{85.1/+}$ (n = 18) flies during a period of light-dark (LD) followed by continuous darkness (DD). The start of the DD period is indicated by a dashed red line at day 5. **f** Rhythmicity (R) and Period (Tau) in control (n = 48) and *Cyfip*$^{85.1/+}$ (n = 45) flies. Two-tailed unpaired Student's t-test, p = 0.272. **g** Night sleep upon *Cyfip* reduction in different clusters of clock neurons or in pars intercerebralis or in ellipsoid bodies. *UAS-Cyfip-IR*$^{1/+}$ (n = 21), *ClkGal4*$^{/+}$ (n = 22), *ClkGal4>UAS-Cyfip-IR*^{1} (n = 16), *Mai179Gal4*$^{/+}$ (n = 23), *Mai179Gal4>UAS-Cyfip-IR*^{1} (n = 23), *CryGal4*$^{/+}$ (n = 23), *CryGal4>UAS-Cyfip-IR*^{1} (n = 34), *pdfGal4*$^{/+}$ (n = 23), *pdfGal4>UAS-Cyfip-IR*^{1} (n = 23), *c819Gal4*$^{/+}$ (n = 22), *c819Gal4>UAS-Cyfip-IR*^{1} (n = 24), *c767Gal4*$^{/+}$ (n = 24), *c767Gal4>UAS-Cyfip-IR*^{1} (n = 24) flies. *UAS-Cyfip-IR*$^{1/+}$ vs. *ClkGal4>UAS-Cyfip-IR*^{1} *p = 0.047; for the other comparisons n.s. = not significant. Mean ± S.E.M. **h** Representative sleep profile in control (n = 49) and *Cyfip*$^{85.1/+}$ (n = 68) flies in basal condition (day 1) and with 6 h sleep deprivation (day 2) and quantification of the Δsleep. Two-tailed Mann-Whitney test, ****p < 0.0001. Mean ± S.E.M. (**a-h**) n.s. = not significant. In (a) and (b) One-way ANOVA

followed by Sidak’s multiple comparison test was used. In (c) and (g) Kruskal-Wallis test followed by Dunn’s multiple comparisons test was used. Source data are provided as a Source Data file.

Supplementary Fig. 4

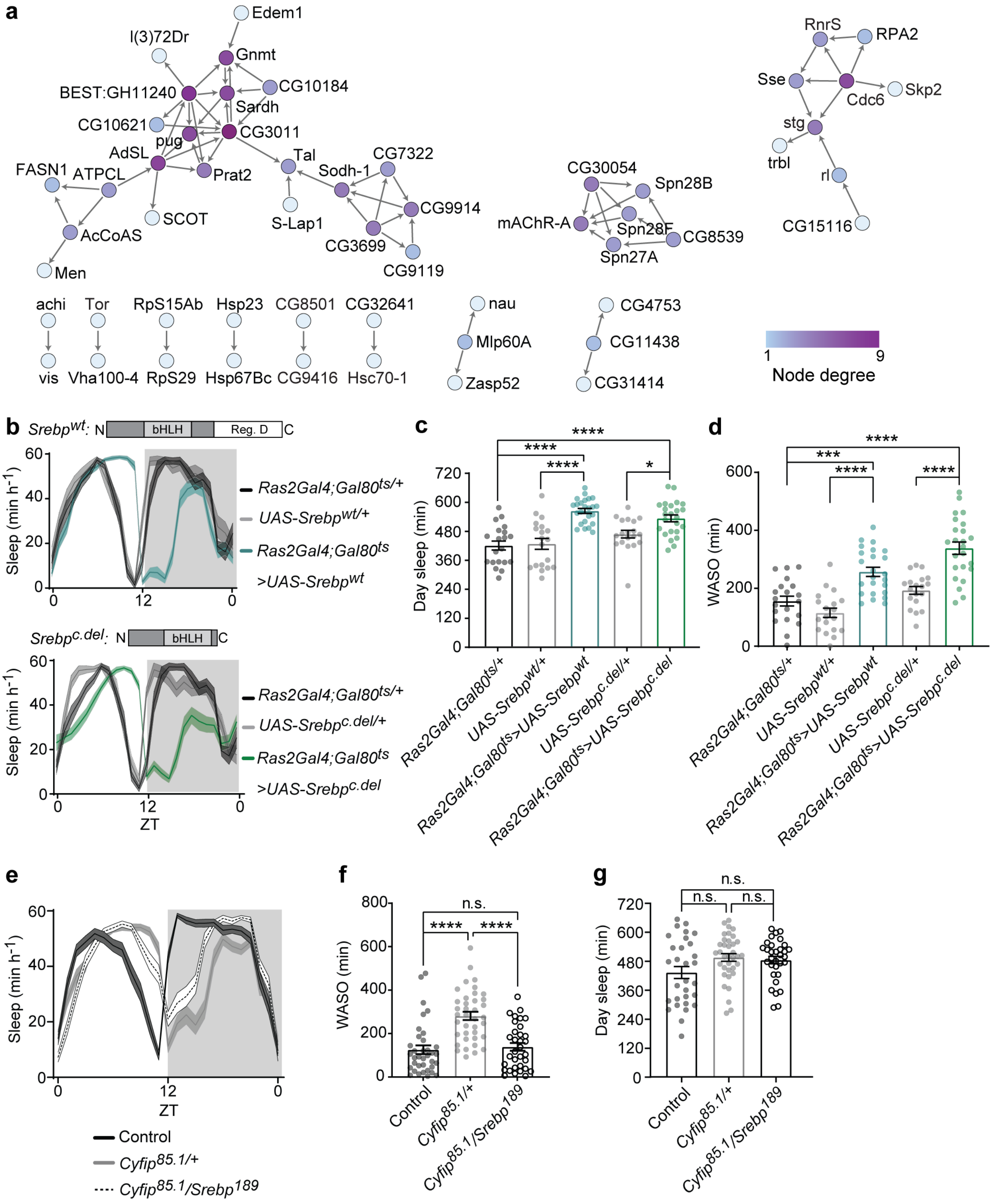

Supplementary Fig. 4 SREBP modulates sleep behavior.

a Protein-protein interaction (PPI) network analysis based on the 130 genes overlapping between *Cyfip*$^{85.1/+}$ mutants and SREBP-OE, using STRING database. Number of interactions (nodes) for each protein is color-coded. **b-d** Sleep profiles (**b**), day sleep (**c**) and WASO (**d**) in *Ras2Gal4;TubGal80*$^{ts/+}$ (*n* = 20), *UAS-Srebp*$^{wt/+}$ (*n* = 19), *Ras2Gal4;TubGal80*^{ts}*>UAS-Srebp*^{wt} (*n* = 24), *UAS-Srebp*$^{c.del/+}$ (*n* = 19) and *Ras2Gal4;TubGal80*^{ts}*>UAS-Srebp*$^{c.del}$ (*n* = 24) flies. One-way ANOVA and Sidak's multiple comparison test: (c) *$p = 0.0209$, ****$p < 0.0001$; (d) ***$p = 0.0003$, ****$p < 0.0001$. Mean ± S.E.M. **e** Representative sleep profiles in control (*n* = 49), *Cyfip*$^{85.1/+}$ (*n* = 46) and *Cyfip*$^{85.1}$*/Srebp*^{189} (*n* = 48) flies. Mean ± S.E.M. **f** WASO in control (*n* = 32), *Cyfip*$^{85.1/+}$ (*n* = 36) and *Cyfip*$^{85.1}$*/Srebp*^{189} (*n* = 32) flies. One-way ANOVA followed by Sidak's multiple comparisons test, n.s. = not significant, ****p < 0.0001. Mean ± S.E.M. **g** Day sleep in control (*n* = 29), *Cyfip*$^{85.1/+}$ (*n* = 36) and *Cyfip*$^{85.1}$*/Srebp*^{189} (*n* = 32) flies. One-way ANOVA followed by Sidak's multiple comparisons test, n.s. = not significant. Mean ± S.E.M. Source data are provided as a Source Data file.

Supplementary Fig. 5

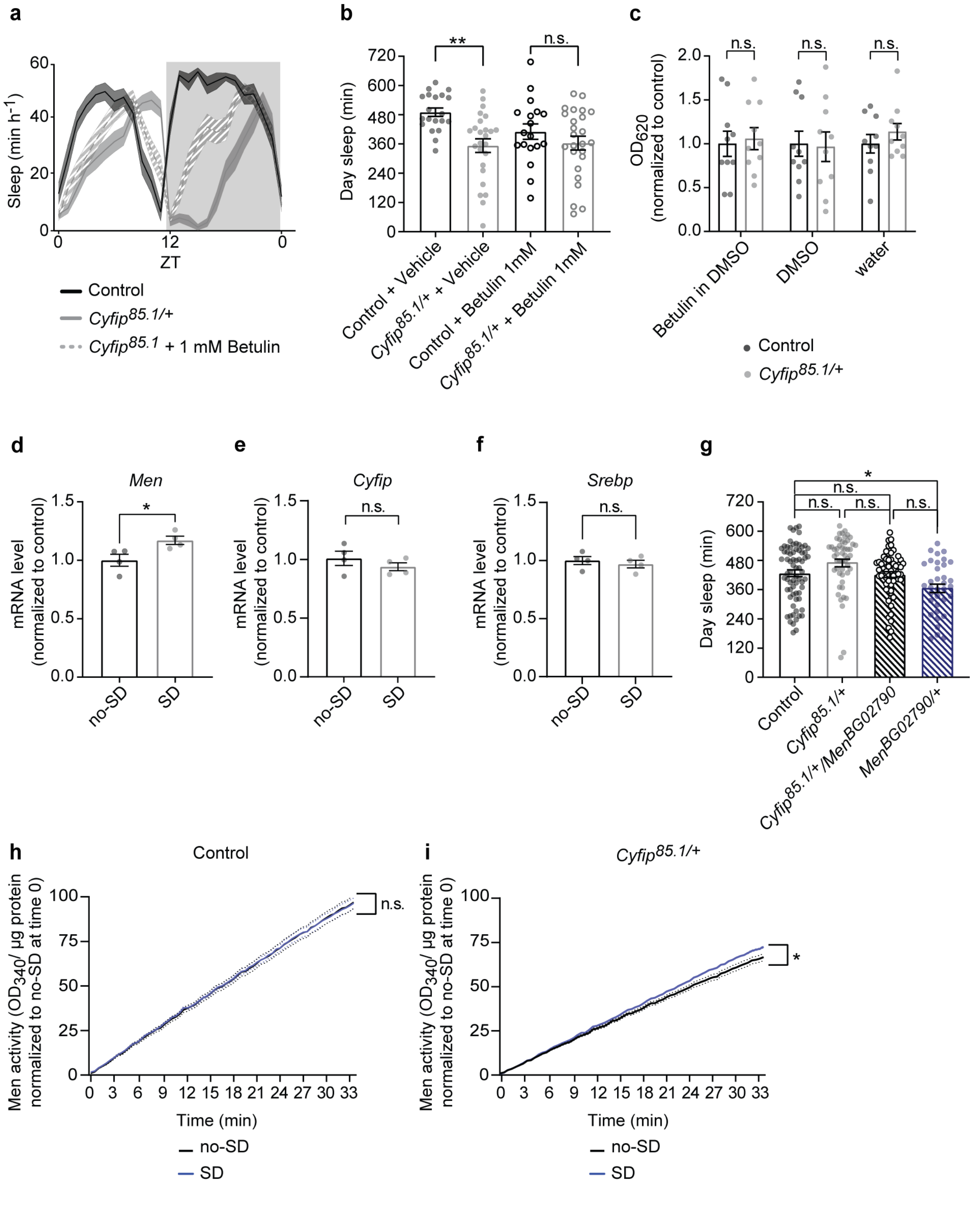

Supplementary Fig. 5 The Men enzyme links SREBP to sleep behavior.

a Representative sleep profile of control and *Cyfip*$^{85.1/+}$ flies treated with 1 mM Betulin or vehicle. Mean ± S.E.M. **b** Day sleep in control (n = 20) and *Cyfip*$^{85.1/+}$ (n = 25) vehicle vs. control (n = 18) and *Cyfip*$^{85.1/+}$ (n = 25) treated flies. Two-way ANOVA and Tukey's multiple comparison test, genotype effect, $p = 0.0004$, $F_{(1, 84)} = 13.57$. Control (vehicle) vs *Cyfip*$^{85.1/+}$ (vehicle) **$p = 0.0012$; for the other comparison n.s. = not significant. Mean ± S.E.M. **c** Quantification of food ingestion in control (n = 10) and *Cyfip*$^{85.1/+}$ (n = 10) flies treated with water, DMSO, or 1mM Betulin in DMSO. Two-way ANOVA. n.s. = not significant. Mean ± S.E.M. **d-f** RT-qPCR in control flies during undisturbed sleep (no-SD, n = 4) and after 8 h of sleep deprivation (SD, n = 4), normalized to *rpl32* mRNA. n = independent samples, pool of 15 fly heads. In (d) *Men*, in (e) *Cyfip*, and in (f) *Srebp* mRNA levels are shown. Unpaired two-tailed Student's t-test, *$p = 0.034$; n.s. = not significant. Mean ± S.E.M. **g** Day sleep in control (n = 71), *Cyfip*$^{85.1/+}$ (n = 51), *Cyfip*$^{85.1}$/*Men*^{BG02790} (n = 60) and *Men*$^{BG02790/+}$ (n = 35) flies. One-way ANOVA and Sidak's multiple comparisons test, control vs. *Men*$^{BG02790/+}$ *$p = 0.039$; for the other comparisons n.s. = not significant. Mean ± S.E.M. **h** Men activity in control no-SD (n = 5) vs. SD (n = 3). n = independent samples, pool of 10 fly heads. Two-way repeated measures ANOVA followed by Sidak's multiple comparisons test, time effect ****$p < 0.0001$, condition effect $p = 0.93$, interaction $p > 0.99$, $F_{(68, 408)} = 0.056$. Control no-SD vs. control SD, n.s = not significant. Mean ± S.E.M. **i** Men activity in *Cyfip*$^{85.1/+}$ no-SD (n = 5) vs. SD (n = 3). n = independent samples, pool of 10 fly heads. Two-way repeated measures ANOVA and Sidak's multiple comparisons test, time effect ****$p < 0.0001$, condition effect $p = 0.107$, interaction ****$p < 0.0001$, $F_{(68, 408)} = 5.266$. *Cyfip*$^{85.1/+}$ no-SD vs. *Cyfip*$^{85.1/+}$ SD *$p = 0.017$. Mean ± S.E.M. Source data are provided as a Source Data file.

Supplementary Table 1

Gene name	Flybase_ID	Gene summary based on the FlyBase Gene Report
AcCoAS	FBgn0012034	Activates acetate so that it can be used for lipid synthesis or for energy generation.
CG10621	FBgn0032726	Predicted to enable S-adenosylmethionine-homocysteine S-methyltransferase activity. Predicted to be involved in S-methylmethionine cycle and methionine biosynthetic process. Is expressed in adult fat body; embryonic/larval crystal cell; extended germ band embryo; and yolk nucleus.
CG16898	FBgn0034480	This gene is referred to in FlyBase by the symbol Dmel\CG16898 (FBgn0034480). Its molecular function is unknown. The biological processes in which it is involved are not known. No phenotypic data is available. The phenotypic class of alleles includes viable.
CG33110	FBgn0053110	Predicted to enable fatty acid elongase activity. Predicted to be involved in fatty acid biosynthetic process and sphingolipid biosynthetic process. Predicted to be integral component of endoplasmic reticulum membrane. Is expressed in adult fat body and embryonic large intestine. Orthologous to human ELOVL1 (ELOVL fatty acid elongase 1) and ELOVL7 (ELOVL fatty acid elongase 7).
CG8539	FBgn0035791	Predicted to enable metallocarboxypeptidase activity. Predicted to be involved in proteolysis. Predicted to be active in extracellular space. Is expressed in adult head and photoreceptor neurons.
CG9837	FBgn0037635	Is expressed in circular visceral muscle fiber; embryonic/larval fat body; embryonic/larval midgut; and germ layer derivative.
Dbi	FBgn0010387	Binds medium- and long-chain acyl-CoA esters with very high affinity and may function as an intracellular carrier of acyl-CoA esters (By similarity). May be involved in energy metabolism in a manner that depends on the substrate used for energy production. Dbi and its metabolites are involved in the regulation of multiple biological processes.
FASN1	FBgn0283427	Fatty acid synthase 1 (FASN1) encodes a fatty acid synthase involved in glycogen metabolism and triglyceride biosynthesis.
Men	FBgn0002719	Malic enzyme (Men) encodes a dehydrogenase that catalyzes the oxidation of malate to pyruvate, with the concomitant reduction of NADP [+] to NADPH. It functions, along with the products of Idh, Zw, and Pgd, in a small network of NADP reducing enzymes.
Prat2	FBgn0041194	Phosphoribosylamidotransferase 2 (Prat2) encodes a type-2 glutamine amidotransferase that is essential in the pathway for de novo synthesis of inosine monophosphate (IMP). IMP is the precursor for purine nucleotides that are required for nucleic acids, energy transfer, cell signaling, and coenzymes.

Supplementary Table 1 Shared genes between *Cyfip*$^{85.1/+}$ and *SREBP*-OE wakefulness-associated genes (WAG).

Comparison of dysregulated WAG between *Cyfip* heterozygous flies and SREBP overexpression[71, 72, 73].

Supplementary Table 2

**Genes**	**Primers (5'-3')**
FASN1 (forward)	GGACGAGGACTATCGCCTTA
FASN1 (reverse)	CTGGATGGTCTTCAGGGACT
FASN3 (forward)	TCCTAACTGTCGCAACATAACA
FASN3 (reverse)	CTCCGGATTAAAATGACGCC
ACC (forward)	ATCGTGGACATTGCTCTTCG
ACC (reverse)	CCAGACCCTCTTTGTGAAGC
Lsd-1 (forward)	TCACAGTCGGCGATAAGTTC
Lsd-1 (reverse)	GTTATCATCCTCCTGTGGGC
Men (forward)	ATTCACCCGACTCTTGGACA
Men (reverse)	TCACGGACCACATAGGGC
AcCoAS (forward)	GGCGACCAGATAGCCTACTA
AcCoAS (reverse)	CAGCAACTTGCGATAGGTGA
ATPCL (forward)	ACTTGCCGTTTTTCCACAGT
ATPCL (reverse)	TAAGTTGATCGGGCTTGCAC
Cyfip (forward)	GATCGCAATGGATTTGTCACG
Cyfip (reverse)	GGAGCACATTCAAGTTGGCAT
Srebp (forward)	GCTCCAAGAGTGCTTGACTG
Srebp (reverse)	GAGCAGTCCTAGTTTCGAGC
Rpl32 (forward)	AGCATACAGGCCCAAGATCG
Rpl32 (reverse)	TGTTGTCGATACCCTTGGGC
Rpl13 (forward)	GTGGTCGAGTTCCGTGAGG
Rpl13 (reverse)	CCTTCTTGGGGTCTCCCTT

Supplementary Table 2 RT-qPCR Primers used in this study.

Gene name, forward, and reverse primers for RT-qPCR are represented in the respective columns.
