# Supplementary material for: SREBP modulates the NADP+/NADPH cycle to control night sleep in Drosophila
Source: Nat Commun. 2023 Feb 20;14:763. doi: 10.1038/s41467-022-35577-8 (PMC9941135; doi:10.1038/s41467-022-35577-8)
Supplement: Supplementary file 2 — Description of Additional Supplementary Files [file 41467_2022_35577_MOESM2_ESM.pdf]

Description of Additional Supplementary Files

Supplementary Data 1. Dysregulated mRNAs in *Cyfip*$^{85.1/+}$ mutants at night-time period. RNA-Seq analysis in the whole fly brain from control and *Cyfip* heterozygous flies (n = 3 independent samples, respectively pool of 20 heads,) at ZT16 revealed a total of 1303 differentially dysregulated genes. Moderated t-test with Benjamini-Hochberg method for p-value adjustment (FDR). Genes with FDR $\leq$ 0.05 and |LFC| $\geq$ 0.585 were considered differentially expressed.

Supplementary Data 2. KEGG enrichment analysis of DEG in *Cyfip*$^{85.1/+}$ flies at ZT16. Kyoto Encyclopaedia of Genes and Genomes (KEGG) pathway enrichment analysis for up- and down-regulated genes (DEG, differentially expressed genes) between control and *Cyfip* heterozygous flies. Only the pathway with Adj. p value $\leq$ 0.1 are presented.

Supplementary Data 3. Wakefulness-associated genes are dysregulated in *Cyfip*$^{85.1/+}$. Upregulated and downregulated genes (FDR $\leq$ 0.05, cut off |LFC| $\geq$ 0.585) in *Cyfip*$^{85.1/+}$ flies have been compared to the database for *Drosophila* wakefulness genes[13].

Supplementary Data 4. Genes shared between the *Cyfip*$^{85.1}$/+ and SREBP overexpression regulon.

Differentially dysregulated genes in *Cyfip* heterozygous flies at ZT16 have been compared with genes dysregulated upon SREBP overexpression[71, 72, 73].

Supplementary Data 5. Wakefulness-associated genes (WAG) regulated by SREBP overexpression.

Genes dysregulated upon SREBP overexpression have been compared with database for *Drosophila* wakefulness-associated genes[13].

Supplementary Data 6. Genotypes and statistics used in this study.

Genotypes, number of independent samples, and descriptive statistics for each figure panel.
