# Supplementary material for: SREBP modulates the NADP+/NADPH cycle to control night sleep in Drosophila
Source: Nat Commun. 2023 Feb 20;14:763. doi: 10.1038/s41467-022-35577-8 (PMC9941135; doi:10.1038/s41467-022-35577-8)
Supplement: Supplementary file 9 — Reporting Summary [file 41467_2022_35577_MOESM9_ESM.pdf]

Corresponding author(s): Claudia Bagni
Last updated by author(s): Dec 6, 2022

Reporting Summary

Nature Portfolio wishes to improve the reproducibility of the work that we publish. This form provides structure for consistency and transparency in reporting. For further information on Nature Portfolio policies, see our Editorial Policies and the Editorial Policy Checklist.

Statistics

For all statistical analyses, confirm that the following items are present in the figure legend, table legend, main text, or Methods section.

n/a	Confirmed	
☐	☒	The exact sample size (*n*) for each experimental group/condition, given as a discrete number and unit of measurement
☐	☒	A statement on whether measurements were taken from distinct samples or whether the same sample was measured repeatedly
☐	☒	The statistical test(s) used AND whether they are one- or two-sided<br>*Only common tests should be described solely by name; describe more complex techniques in the Methods section.*
☐	☒	A description of all covariates tested
☐	☒	A description of any assumptions or corrections, such as tests of normality and adjustment for multiple comparisons
☐	☒	A full description of the statistical parameters including central tendency (e.g. means) or other basic estimates (e.g. regression coefficient) AND variation (e.g. standard deviation) or associated estimates of uncertainty (e.g. confidence intervals)
☐	☒	For null hypothesis testing, the test statistic (e.g. *F*, *t*, *r*) with confidence intervals, effect sizes, degrees of freedom and *P* value noted<br>*Give P values as exact values whenever suitable.*
☒	☐	For Bayesian analysis, information on the choice of priors and Markov chain Monte Carlo settings
☒	☐	For hierarchical and complex designs, identification of the appropriate level for tests and full reporting of outcomes
☒	☐	Estimates of effect sizes (e.g. Cohen's *d*, Pearson's *r*), indicating how they were calculated

Our web collection on statistics for biologists contains articles on many of the points above.

Software and code

Policy information about availability of computer code

Data collection	Detailed description of data collection can be found in the methods section.
Data analysis	Analysis of sleep behavior was done using a custom R (v. 4.2.1) script (available at https://github.com/adrianclo/dam3) and FaasX (v1.21). Analysis of WB was performed using GE Health's ImageQuant TL (v.8.1). Analysis of fluorescence intensity was performed using ImageJ (v1.53c). Statistics were performed using Graphpad Prism (v.9) and R (v.4.2.1).<br>RNA-Seq data processing and Analysis: Cutadapt (v. 1.8), fastq_screen (version. 0.9.3), reaper (version. 15-065), STAR (version. 2.5.2b, RSEM (version. 1.2.31), htseq-count (version. 0.6.1) using Drosophila melanogaster.BDGP6.86 gene annotation. Quality of the RNA-Seq data alignment was assessed using RSeQC (version. 2.3.7). Statistical analysis was performed for genes in R (R version 3.3.2), TMM normalization (EdgeR package version 3.14.0118) and limma voom function (Limma package version 3.28.21). KEGG pathways analysis for the DEGs was performed using GeneTrail2 3.0 (https://genetrail2.bioinf.uni-sb.de).<br>PPI Networks Analysis was performed using the STRING interactome (https://string-db.org) and visualized using Cytoscape 3 (RRID:SCR_003032).<br>Raw LC-MS/MS data were processed using the Agilent Quantitative analysis software (version B.07.00, MassHunter Agilent technologies) and signal intensity drift correction and noise filtering were compiled within the MRM PROBS software.

For manuscripts utilizing custom algorithms or software that are central to the research but not yet described in published literature, software must be made available to editors and reviewers. We strongly encourage code deposition in a community repository (e.g. GitHub). See the Nature Portfolio guidelines for submitting code & software for further information.

Data

Policy information about availability of data

All manuscripts must include a data availability statement. This statement should provide the following information, where applicable:

- Accession codes, unique identifiers, or web links for publicly available datasets
- A description of any restrictions on data availability
- For clinical datasets or third party data, please ensure that the statement adheres to our policy

All relevant data are available in the manuscript, in Supplementary Information files and/or are provided as Source Data. The RNA-Seq data generated in this study will be deposited in the GEO (NCBI) database under accession code: GSE220524. Further information and requests for resources and reagents should be directed to and will be fulfilled by the Lead Contact, Prof. Claudia Bagni (claudia.bagni@unil.ch). There are restrictions to the availability of Cyfip mutant flies due to a signed MTA with Dr. Angela Giangrande (CNRS, Université de Strasbourg, France).
Databases/Datasets used in the text comes from: Flybase, DRSC/TRiP Functional Genomic Resources and dataset from the following 4 publications:
1) Cirelli C et al., (2005). Sleep and wakefulness modulate gene expression in Drosophila. DOI: 10.1111/j.1471-4159.2005.03291.x.
2) Horton JD, et al. (2003). Combined analysis of oligonucleotide microarray data from transgenic and knockout mice identifies direct SREBP target genes. DOI: 10.1073/pnas.1534923100.
3) Rome S, et al. (2008). Microarray analyses of SREBP-1a and SREBP-1c target genes identify new regulatory pathways in muscle.DOI: 10.1152/physiolgenomics.90211.2008.
4) Tsai JW, et al. (2019) Transcriptional Feedback Links Lipid Synthesis to Synaptic Vesicle Pools in Drosophila Photoreceptors. DOI: 10.1016/j.neuron.2019.01.015.

Field-specific reporting

Please select the one below that is the best fit for your research. If you are not sure, read the appropriate sections before making your selection.

☒ Life sciences ☐ Behavioural & social sciences ☐ Ecological, evolutionary & environmental sciences

For a reference copy of the document with all sections, see nature.com/documents/nr-reporting-summary-flat.pdf

Life sciences study design

All studies must disclose on these points even when the disclosure is negative.

Sample size	Sample size was chosen based on published literature of Drosophila and sleep behavior (DOI: 10.1038/s41586-019-1034-5; DOI: 10.1126/science.287.5459.183; DOI:10.1038/s41586-021-03954-w). Sample size for metabolomic and colorimetric analysis was chosen based on published literature (DOI: 10.1016/j.cell.2020.02.044). In all cases the samples size was chosen to satisfy statistical power.
Data exclusions	Flies that were not moving (total amount of sleep > 705/720 minutes, total of the sleep amount in a single night) or died at the end of the experiment, were excluded from the analysis. In the experiment in Figure 1d, flies that were not responding to light stimuli with a reduction of sleep > 2% of the baseline were excluded from the analysis. Outliers as defined by the Grubbs' test for outliers were removed, although in the majority of the experiments we did not have outliers.
Replication	Data were obtained from multiple independent biological samples. The number of independent biological replicates is stated in the figure legend. RNA-seq, HILIC-MS/MS, colorimetric and enzyme activity experiments were performed with at least n = 3 independent biological samples.
Randomization	Samples were allocated based on the corresponding genotype. In experiments were the same genotype was used in different conditions (noSD vs SD; veh vs. treated) flies of the same progeny were randomly allocated to the different experimental groups.
Blinding	For most of the experiment the experimenters were blind to the genotype during data collection and analysis. Sleep behavior experiments were not generally blind to fly genotype because data collection and analysis is automated and therefore not subjected to experimenter bias. Confocal microscopy pictures analysis were conducted blind with regards to the genotype.

Reporting for specific materials, systems and methods

We require information from authors about some types of materials, experimental systems and methods used in many studies. Here, indicate whether each material, system or method listed is relevant to your study. If you are not sure if a list item applies to your research, read the appropriate section before selecting a response.

Materials & experimental systems

n/a	Involved in the study
☐	☒ Antibodies
☒	☐ Eukaryotic cell lines
☒	☐ Palaeontology and archaeology
☐	☒ Animals and other organisms
☒	☐ Human research participants
☒	☐ Clinical data
☒	☐ Dual use research of concern

Methods

n/a	Involved in the study
☒	☐ ChIP-seq
☒	☐ Flow cytometry
☒	☐ MRI-based neuroimaging

Antibodies

Antibodies used

anti-SREBP (BD Biosciences, cat. 557036, RRID: AB_384985), anti-Syntaxin (DHSB, cat. 8c3, RRID: AB_528484), anti-GFP (Invitrogen, cat. A-11122, RRID: AB_221569), anti-BRP (cat. nc82, DSHB, RRID: AB_2314866), anti-HA 11 epitope tag (clone 16B12) (BioLegend, cat. 901501, RRID: AB_2801249) - Alexa Fluor (Thermo Fisher Scientific): anti-rabbit IgG 488 (cat. A-11034, RRID: AB_2576217), anti-mouse IgG 488 (cat. A-11029, RRID: AB_2534088), anti-mouse IgG 546 (cat. A-11030, RRID: AB_2534089), anti-mouse IgG 647 (cat. A-21236, RRID: AB_2535805), mouse 680- (cat. 35519, RRID: AB_ AB_1965956) and 800- (cat. SA5-10176, RRID: AB_2556756) DyLight (ThermoScientific). Concentrations of the antibodies is reported in Methods.

Validation

Antibodies used in this study were commercially available, previously validated and described in published work and/or by manufacturer:
anti-SREBP: doi: 10.1016/j.celrep.2017.09.089;

anti-Syntaxin 8c3: doi: 10.1083/jcb.201108088; doi: 10.1016/j.cell.2005.03.012 and many others reported in DHSB webpage: https://dshb.biology.uiowa.edu/8C3;

anti-BRP (nc82): doi:10.1126/science.1166673; doi: 10.1016/j.cell.2009.08.034 and many others reported in the DHSB webpage: https://dshb.biology.uiowa.edu/nc82;

anti-HA (901501): https://doi.org/10.1016/j.celrep.2022.110517, https://doi.org/10.1083/jcb.201803099.

anti-GFP (A-11122): https://doi.org/10.1016/j.neuron.2021.07.006; https://doi.org/10.7554/eLife.30327;

Alexa Fluor anti-rabbit IgG 488 (cat. A-11034, RRID: AB_2576217), validated by manufacturer, https://www.thermofisher.com/antibody/product/Goat-anti-Rabbit-IgG-H-L-Highly-Cross-Adsorbed-Secondary-Antibody-Polyclonal/A-11034.

Alexa Fluor anti-mouse IgG 488 (cat. A-11029, RRID: AB_2534088), validated by manufacturer, https://www.thermofisher.com/antibody/product/Goat-anti-Mouse-IgG-H-L-Highly-Cross-Adsorbed-Secondary-Antibody-Polyclonal/A-11029.

Alexa Fluor anti-mouse IgG 546 (cat. A-11030, RRID: AB_2534089),validated by manufacturer, https://www.thermofisher.com/antibody/product/Goat-anti-Mouse-IgG-H-L-Highly-Cross-Adsorbed-Secondary-Antibody-Polyclonal/A-11030.

Alexa Fluor anti-mouse IgG 647 (cat. A-21236, RRID: AB_2535805), validated by manufacturer, https://www.thermofisher.com/antibody/product/Goat-anti-Mouse-IgG-H-L-Highly-Cross-Adsorbed-Secondary-Antibody-Polyclonal/A-21236;

DyLight mouse 680- (cat. 35519, RRID: AB_ AB_1965956) ,validated by manufacturer, https://www.thermofisher.com/antibody/product/Goat-anti-Mouse-IgG-H-L-Cross-Adsorbed-Secondary-Antibody-Polyclonal/35519.

Dylight mouse 800- (cat. SA5-10176, RRID: AB_2556756) ,validated by manufacturer, https://www.thermofisher.com/antibody/product/Goat-anti-Mouse-IgG-H-L-Cross-Adsorbed-Secondary-Antibody-Polyclonal/SA5-10176.

Animals and other organisms

Policy information about studies involving animals; ARRIVE guidelines recommended for reporting animal research

Laboratory animals

This study is based on different transgenic strains of male Drosophila melanogaster. These were mostly obtained from Bloomington Drosophila Stock Center or from colleagues of the Drosophila scientific community. The fly strains carrying the different transgenes or genomic mutations are indicated in Methods section:

-The following fly stocks were gifts from:
Cyfip85.1 and UAS-Cyfip-IR1 (Dr. A. Giangrande, IGBMC - France);
Ras2Gal4;TubGal80ts, NrvGal4 and Gad1Gal4 (Dr. E.M.C. Skoulakis, BSRC - Greece);
R23E10Gal4 (Dr. G. Miesenbock, CNCB - UK);
RepoGal4 (Dr. L. Neukomm, UNIL - Switzerland)
ClkGal4_4.1M, Mai179Gal4 and CryGal4_39 (Dr. F. Rouyer, CNRS - France);
APLGal4 (VT043924-GAL4) (Dr. A. Fiala, UG - Germany);

-Fly lines obtained from the Bloomington Stock Center (BDSC):
w1118 (BDSC 3605); Srebp189 (39396), GAL4-dSREBPg.K-B31:UAS-GFP ('SREBP activation reporter line', 39612), UAS-SrebpWT (8236), UAS-Srebpc.del (constitutively active Srebp, 8244), MenBG02790 (12824), AralarMI07552 (43727), PdfGal4 (6900), c819Gal4 (30849), c767Gal4 (30848) and Herm3xP3-ECFP,alphatub-piggyBacK10_M6 (32070).

-The UAS-Cyfip-IR2 (37908) line was obtained from the Vienna Drosophila Resource Center.

-The following stock was created in this work: w1118;;CYFIP-N-HA;

Wild animals	No wild animals were used in this study.
Field-collected samples	No field collected data were used in this study.
Ethics oversight	No ethical approval is required to work with Drosophila.

Note that full information on the approval of the study protocol must also be provided in the manuscript.
